# Increased Pay for Health Officers

**Published:** 1911-07

**Authors:** 


### Increased Pay for Health Officers

When the budget for the coming fiscal year was presented to Mayor Fuhrmann, of Buffalo, for action he vetoed, among other items, the salary increases for the officials of the health department. At the meeting of the board of councilmen on June 21 the increases were passed over the veto. During the debate incident to the action there was considerable criticism of the mayor's action much of it ill-advised and nearly all of it brought forth by the impression that in vetoing the increases the mayor had in mind a purely perfunctory reduction in the city tax rate. While this may have been the objective point the veto was an error of administrative judgment and it is well that the board of councilmen, usually deficient in matters of public policy of importance, should have over ridden the mayor and given to this department, one of the most vitally important of the city, adequate pay for the excellent service it is rendering.

The increases granted raise the salary of the commissioner from $4,000 to $5,000 and of the assistant commissioner from $2,000 to $3,000. Other increases go to the chemist, the bacteriologist and his two assistants and the employees in the laboratory department and to the chief inspector of plumbing.

